# “Deconstructing” Scientific Research: A Practical and Scalable Pedagogical Tool to Provide Evidence-Based Science Instruction

**DOI:** 10.1371/journal.pbio.1000264

**Published:** 2009-12-22

**Authors:** Ira E. Clark, Rafael Romero-Calderón, John M. Olson, Leslie Jaworski, David Lopatto, Utpal Banerjee

**Affiliations:** 1 Department of Molecular, Cell, and Developmental Biology, University of California Los Angeles, Los Angeles, California, United States of America; 2 Department of Psychology, Grinnell College, Grinnell, Iowa, United States of America; 3 Department of Biological Chemistry; Molecular Biology Institute; Broad Stem Cell Research Center, University of California Los Angeles, Los Angeles, California, United States of America; University of California Berkeley/JGI, United States of America

## Abstract

Focused analysis of current research projects provides an effective platform for teaching early-stage undergraduates the logic of scientific inquiry.

There is growing interest among scientists and science educators to include active learning approaches that allow students to appreciate how primary evidence is used to construct scientific knowledge [1],[2]. Indeed, the National Academies and others have recognized four essential objectives for science education at elementary, middle and high school, and undergraduate levels: (1) understanding and utilizing scientific explanations of the natural world, (2) knowing how to generate and evaluate scientific evidence, (3) understanding the nature and development of scientific knowledge, and (4) participating productively in scientific practices and discourse [2]–[5]. In the life sciences, both discovery-based research courses and journal clubs accomplish many of these learning goals with undergraduates [6]–[10], although each has significant limitations. Hands-on research classes have proven to be a successful entry point for training new students in the process of scientific discovery, but, with the exception of bioinformatics-based classes [10], the heavy demand for space and resources constrains the scalability of these strategies. Journal clubs are logistically easier to run, but are only effective in small formats and are usually limited to more advanced students.

To address these issues, we have designed a strategy we call “research deconstruction” that trains first- and second-year undergraduates to analyze real data from current, cutting-edge research, presented to them in the form of a high-level research seminar. We teach the deconstruction course in two five-week modules, each module beginning with an hour-long, full-scale research seminar by an invited faculty speaker. At this point, the students have at best a rather superficial comprehension of the seminar, as we encourage the speaker to deliver his or her standard research presentation, replete with experimental data normally presented to a more sophisticated audience. A separate course instructor then distils the content of the seminar over 10 contact hours of classroom instruction. As the research seminar is videotaped and archived, students can refer back to it regularly. Each classroom lecture typically focuses on approximately 5–10 minutes of the seminar, allowing the instructor to approach each fragment independently from many different angles and explore the fundamental concepts underlying the creation of the data. (For examples of seminar excerpts and their deconstruction, see Videos S1, S2, and S3).

During the deconstruction phase, the students identify hypotheses from the seminar, explore the experimental approaches used, and actively analyze the data—a collective exercise that deconstructs a complex research seminar into manageable portions. As concepts and techniques are introduced to them, stripped of jargon, the students begin to see the logic of the research. In the process, they follow the story of the seminar and experience discovery moments as the implications of each experiment become clear.

Consistent with the four above-mentioned objectives for science education [1]–[5], we require our students to independently scrutinize data and generate valid conclusions. Class assignments avoid testing memorization of facts in favor of testing the ability to formulate novel hypotheses, propose experiments, and suggest future directions for the research. (See Text S1 for sample problem set questions). Ample office hours are made available throughout the course for students to discuss any conceptual problems that may arise.

Remarkably, by the end of the five-week period, students are able to discuss the experiments intelligently and critically, and can apply the techniques they learned to hypothetical scenarios involving scientific research within as well as outside the field of the seminar presentation. This is further evidenced at an hour-long question and answer session hosted by the seminar speaker at the end of the module. While students are generally reluctant to ask questions when they first hear the seminar, by the end of the deconstruction they have the confidence to engage the speaker and ask thoughtful and often challenging questions. Speakers have commented favorably on the level of discussion in the Q&A sessions and the improvement they perceive in student comprehension over the five weeks since they presented their research. (See excerpts of faculty testimonials in Figure 1 and more extensive comments in Text S2).

**Figure 1:**
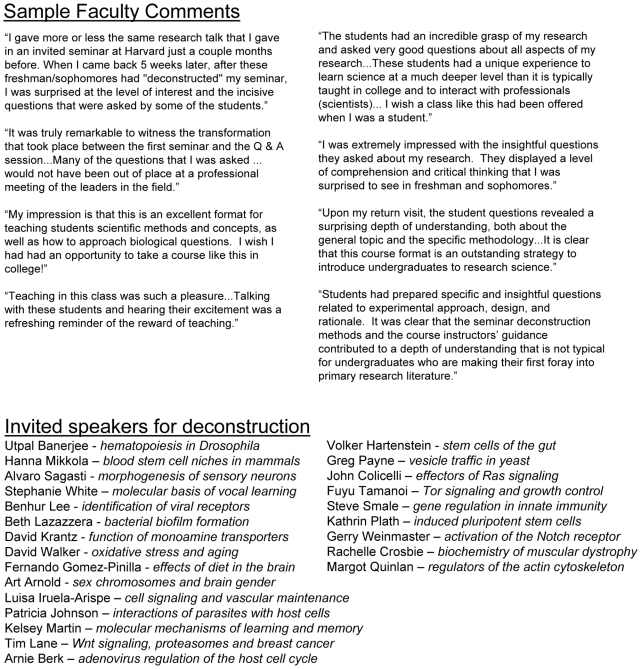
Excerpts of comments from invited faculty speakers and research topics deconstructed. These comments should be viewed only as testimonials and not as data. For more complete impressions, see Text S2. Names and seminar topics of faculty speakers who have participated in the research deconstruction courses from Spring 2007–Spring 2009.

No laboratory infrastructure is necessary for this methodology, and the seminar deconstruction format is readily adapted to a variety of subjects and scientific disciplines. To date, 24 different faculty members have participated in the courses, presenting research on a wide range of topics including stem cell biology, epigenetics, neurobiology, and microbiology (Figure 1). We have received enthusiastic participation by our strongest research faculty, who have recognized that by delivering their current research seminar and hosting the final Q&A session, they provide a valuable and effective bridge between their research and educational efforts, offering large numbers of students the opportunity to engage directly in diverse fields of scientific study. The research deconstruction approach is comparable to hands-on research courses in teaching students to evaluate and interpret scientific evidence, while at the same time being highly scalable and easily transferable to other institutions. Over seven academic quarters at University of California Los Angeles (UCLA), we have used this strategy to train almost 500 undergraduates from a variety of majors, most of whom are first- and second-year students with minimal preparation in the life sciences.

We have previously described our Howard Hughes Medical Institute (HHMI)–funded hands-on research program, the Undergraduate Research Consortium in Functional Genomics (URCFG), which over the past six academic years has trained nearly 500 students in scientific discovery through direct participation in original research [6],[8]. By several criteria, URCFG has been quite successful. The program has yielded several peer-reviewed publications, including two papers with 134 and 264 undergraduate authors [6],[8],[11],[12]. It has identified students for further independent research, many of whom have since graduated and are now in Ph.D. or M.D.-Ph.D. programs. Finally, survey data indicate that students in URCFG report significant gains in a number of important areas such as understanding science, analyzing data and interpreting results ([8] and Figure 2).

**Figure 2:**
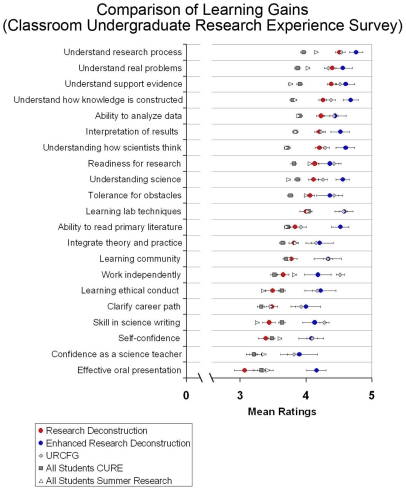
Learning gains produced by UCLA research deconstruction and hands-on research (URCFG) courses. CURE survey data from Spring 2007–Spring 2009 are compared to the means from all students participating in the CURE survey during Spring 2009, as well as to students engaged in a summer research experience in 2008, as measured by the comparable SURE II (Summer Undergraduate Research Experience) survey. The CURE and SURE surveys include identical items that permit comparisons. The CURE reference cohort derived from introductory to advanced biology courses that contained some research-related component. The typical student in the SURE cohort was a third- or fourth-year student. Scale: 1 = little to no gain; 2 = small gain; 3 = moderate gain; 4 = large gain; 5 = very large gain. Average N values: UCLA research deconstruction – 157; UCLA enhanced research deconstruction – 24; URCFG – 147; all students CURE – 598; all students summer research – 1,489. Error bars represent one standard error.

Assessment data from the Classroom Undergraduate Research Experience (CURE) survey ([13],[14] and http://www.grinnell.edu/academic/psychology/faculty/dl/sure&cure/) show that students from the research deconstruction course report learning gains as high as or greater than those of reference cohorts, including students engaged in a summer research experience, in nearly all areas surveyed (Figure 2). The learning gains are not as strong in some areas as those reported by URCFG students, which are considerably better than those of the reference cohorts in all skills except oral presentation (an element not emphasized in URCFG). However, in several important areas, including understanding the research process, how knowledge is constructed, and the role of supporting evidence, learning gains reported by students of the deconstruction courses compare favorably with those of URCFG students and are considerably better than those of reference cohorts. Thus, exposing students within a classroom setting to the design and execution of a research project appears to be an effective means of teaching them the logic of research.

To further improve upon the learning gains from research deconstruction, we have created an “enhanced” version of the course, taught to a smaller group of students from the larger research deconstruction course or from URCFG. Students are accepted into the enhanced course based on their interest in research and performance in the previous course. The enhanced research deconstruction course includes assignments of primary literature, student presentations of research papers, written reports on the research seminars, and a strong emphasis on experimental design and proper use of controls (for an example of the enhanced research deconstruction delivered to students who have previously taken the basic course, see Video S4). Early indications from the CURE survey suggest that these changes yield learning gains comparable to or better than URCFG in almost all areas measured (Figure 2). The improvements observed may result from elements added to the course syllabus, smaller class size, student selection, benefit of a prior experience in evidence-based analysis, or, most likely, a combination of these factors. We conclude that a combination of a regular and an enhanced deconstruction experience elicits the highest gains for the student. However, we emphasize that even the basic deconstruction course alone is effective at eliciting gains in important conceptual areas that are vital to science education.

The deconstruction format has been valuable in identifying students with promise for productive independent research. Like URCFG, it serves as a screening course to recruit students for the newly created UCLA Minor in Biomedical Research (http://www.biomedresearchminor.ucla.edu), a comprehensive research training program that places promising students in laboratories throughout the College and the School of Medicine while providing didactic training to complement their research. Since the spring of 2007, the larger deconstruction classes have placed 79 students within this minor, compared to 43 from URCFG, which is limited in scale due to the demand for laboratory resources.

Previous studies have shown that analysis of primary research literature is a highly effective way to train students in understanding how knowledge is created and evidence evaluated [7],[15]. Scientific instruction in the context of real research problems may be comparable to use of case studies in promoting higher order critical thinking [16]. Our experience suggests that an extensive theoretical knowledge base is not essential for early-stage undergraduates to understand biomedical research. In fact, the research deconstruction course format emulates the scientific process, whereby students begin by analyzing data, and end by using it to derive and appreciate general biological principles. A valuable component to add to the deconstruction approach may be seen in the use of adapted primary literature (APL), a format designed for high school students, derived from primary research papers [17],[18].

Research deconstruction provides an effective pedagogical tool to offer evidence-based science instruction to a large number of early-stage students. Demanding very few material resources, it is a strategy that can be adopted by a broad spectrum of academic institutions. For the future, research seminars available from Internet resources, such as the American Society for Cell Biology's iBioSeminars (http://www.ibioseminars.org), might also be used as a resource for material to deconstruct in the classroom. A Web-based repository of both seminars and deconstruction classes that is updated on a regular basis will also prove to be a valuable resource that can be accessed universally for use in any course.

## Supporting Information

Text S1 **Sample problem set questions from research deconstruction courses.**
(0.03 MB DOC)

Text S2 **Comments from invited faculty speakers who participated in the research deconstruction courses, provided as testimonials.**
(0.03 MB DOC)

Video S1 **Video excerpts of seminar and deconstruction classes.**
(18.04 MB MOV)

Video S2 **Video excerpts of seminar and deconstruction classes.**
(17.29 MB MOV)

Video S3 **Video excerpts of seminar and deconstruction classes.**
(17.11 MB MOV)

Video S4 **Video excerpt of seminar and enhanced deconstruction class.**
(14.03 MB MOV)
