# Supplementary material for: “Deconstructing” Scientific Research: A Practical and Scalable Pedagogical Tool to Provide Evidence-Based Science Instruction
Source: PLoS Biol. 2009 Dec 22;7(12):e1000264. doi: 10.1371/journal.pbio.1000264 (PMC2796859; doi:10.1371/journal.pbio.1000264)
Supplement: Text S1 — Sample problem set questions from research deconstruction courses. (0.03 MB DOC) [file pbio.1000264.s001.doc]

**Text S1**

**Example Problem Set Questions**

The Colicelli lab used Western blots with an anti-phosphotyrosine antibody to determine whether CRK and CRKL are phosphorylated. When they tested phosphorylation of CRK in an experiment with only purified proteins, they just used a Western blot. But when they wanted to test phosphorylation of CRKL in cells, they did an immunoprecipitation with anti-CRKL antibody before doing the Western with the anti-phosphotyrosine antibody. Why did they need to do an immunoprecipitation first?

There is a bacterial protein that has only one WH2 domain but is able to nucleate actin. Based on Dr. Quinlan’s proposed mechanism for actin assembly by the WH2 domains of Spir, would you expect a protein that contains only a single WH2 domain to be capable of nucleating actin filaments? What additional property might be required for such a protein to nucleate actin?

A biotech company discovers a chemical compound, cleverly named Compound A, that binds to β-catenin. When tissue culture cells are treated with Compound A, β-catenin levels appear to increase, but no β-catenin protein is detectable in the nucleus. Somehow Compound A interferes with both the degradation of β-catenin and its ability to enter the nucleus.

How might this compound affect Wnt signaling? What do you think might be the effect of such a drug on colon cancers caused by mutation of the APC gene (adenomatous polyposis coli), which encodes a protein necessary to degrade β-catenin? Explain your reasoning. (*assigned during deconstruction of research presented by Timothy Lane*)

Why was support bone marrow used in the transplantation experiments of Dr. Mikkola? How could she distinguish blood cells derived from placental HSCs from blood cells derived from the support bone marrow?

Why was it important for the Iruela-Arispe lab to knock out the VEGF gene only in the endothelial cells, as opposed to the entire animal?

Why can processing of alpha-factor be used as an assay for defects in TGN-endosome trafficking? (*assigned during deconstruction of research presented by Greg Payne*)

You have immunoprecipitated an unknown membrane protein you think is the receptor of the South Pacific virus. You run a Western blot and get three distinct bands of varying sizes. Can you think of two different hypotheses that would account for this result? What experiments would you design to determine which of these two hypotheses is correct? (*assigned during deconstruction of research presented by Benhur Lee*)

Dr. Walker’s research clearly showed that overexpression of *Glaz* in *Drosophila* S2 cells protects against Aβ-42 toxicity (slide #25). However, the experiment failed to show whether it was the intracellular or extracellular *Glaz* that provides the protection. How could you determine which *Glaz* (intra or extracellular) specifically confers resistance against Aβ-42 cytotoxicity?

You are reviewing a manuscript for a respected journal. One of the experiments you go over uses BrdU to identify actively dividing cells. The authors are pulsing BrdU to their flies for 1, 3 and 5 days and then immediately stain for BrdU incorporation (no chase). They see no labeling of nuclei and so they conclude that there are no stem cells in the tissues they look at. In your review letter to the editor, you decide to comment on this experiment and conclusion. In particular, you are concerned that the conclusions are not appropriate. What do you think is wrong with the authors’ conclusions? What would you suggest they do to make their conclusions stronger? (*assigned during deconstruction of research presented by Volker Hartenstein*)

A colleague of yours discovers a new species of zebrafish from the north-eastern region of the Himalayas. She decides to perform ablation experiments on this fish, and removes the trigeminal ganglion early in development on one side of the head. She notices that the peripheral axons of the neurons on the intact side stop at the midline anyway. She concludes that in this new species, the inhibitory signals are secreted by some unknown cell type at the midline. Being a good scientist, you decide to give her an alternative hypothesis. What could it be? (*assigned during deconstruction of research presented by Alvaro Sagasti*)
