# Supplementary material for: “Deconstructing” Scientific Research: A Practical and Scalable Pedagogical Tool to Provide Evidence-Based Science Instruction
Source: PLoS Biol. 2009 Dec 22;7(12):e1000264. doi: 10.1371/journal.pbio.1000264 (PMC2796859; doi:10.1371/journal.pbio.1000264)
Supplement: Text S2 — Comments from invited faculty speakers who participated in the research deconstruction courses, provided as testimonials. (0.03 MB DOC) [file pbio.1000264.s002.doc]

**Text S2**

**Faculty Comments**

“I have given two research presentations, with subsequent discussion classes, to 5HB students. Both times I was extremely impressed with the insightful questions they asked about my research. They displayed a level of comprehension and critical thinking that I was surprised to see in freshman and sophomores.”

“Last year, I had the pleasure and honor of participating in BR 5HB as a guest lecture, speaking to a class of about 15 freshman and sophomores. The students were a bit overwhelmed by my talk, as expected. One student did ask a good scientific question that day but this was in stark contrast to when I returned for the Q&A session a few weeks later. The students had an incredible grasp of my research and asked very good questions about all aspects of my research. They also took advantage of the time to ask me about why I chose to do basic science research as opposed to more directly medically related work and to ask about my career choice and experience. These students had a unique experience to learn science at a much deeper level than it is typically taught in college and to interact with professionals (scientists). I benefitted directly from this experience in two ways. First, I got feedback on my work from a new audience with a fresh perspective. Second, one of the students was interested in my research and joined my lab. He is now a productive member of my lab. I wish a class like this had been offered when I was a student.”

“5HA was a remarkable experience. I gave more or less the same research talk that I gave in an invited seminar at Harvard just a couple months before. When I came back 5 weeks later, after these freshman/sophomores had "deconstructed" my seminar, I was surprised at the level of interest and the incisive questions that were asked by some of the students.  This course appears to be a novel and unusual way of exposing young students to the world of real biomedical research.”

“I enjoyed presenting my initial lecture to the class, as well participating in the follow-up session after my lecture had been deconstructed. I was very impressed with the sophistication of the students and the questions that they asked in both sessions, and in particular, the follow-up session. I think that you have developed a terrific way to learn how to appreciate scientific seminars and to understand the way the science really works. I wish that a similar model had been in place when I was a student, struggling to understand anything that seminar speakers would say after the first one or two introductory slides.”

“I had an incredibly rewarding and interesting experience taking part in the BR 5HA seminar class.  It was truly remarkable to witness the transformation that took place between the first seminar and the Q & A session.  In just a few short weeks, these bright young students became world experts in some fairly sophisticated molecular genetic tools and how these tools can be used to uncover the molecular mechanisms underlying the aging process.  Many of the questions that I was asked in the second seminar would not have been out of place at a professional meeting of the leaders in the field.  I have no doubt that some of the students from this class will one day be scientific leaders presenting their own work to a bunch of enthusiastic undergrads. When this happens, I hope that they enjoy it as much as I did.”

“I was impressed that I was asked to give my 'normal seminar' rather than to simplify it for the audience of freshman and sophomores. I did so and expected the students to be both confused and shy, but they asked excellent questions right away. In fact, we had so much discussion that I partially lost my voice. But the next Q&A session was what truly impressed me. It was clear that the students had absorbed the content of my work and were ready to challenge me with new takes on it and new ideas for experiments. The 'deconstruction' idea is very innovative, exciting, and I believe, effective.”

“I presented a seminar in the MCDB “seminar deconstruction” course last year on my lab’s investigations into .... As instructed, I did not simplify the seminar for the students. The talk I gave was typical of one that I would give at a scientific meeting and included embryological, genetic, and molecular experiments. During the seminar itself the students were attentive and inquisitive, but the value of the course was most apparent when I returned for the question-and-answer session. The students had no shortage of questions and comments, ranging from the technical to the conceptual. Some of these comments were quite sophisticated, demonstrating not only a clear understanding of the experiments themselves, but also a thoughtful consideration of their implications. Many of the questions were similar to those I am typically asked by experienced scientists in the field. My impression is that this is an excellent format for teaching students scientific methods and concepts, as well as how to approach biological questions. I wish I had had an opportunity to take a course like this in college!”

“I do like the idea of the class - as it is set up for the undergrad to understand the details of an experiment, why it was done, what the tools are the address the question and how to evaluate the data. I was impressed by the questions I got in the Q and A session which demonstrated that that undergrads indeed understood the problem and the data and that they could come up with follow up questions. I do believe that this course is extremely powerful in exciting young people for science.”

“My experience in LH5B was very enjoyable and stimulating - this is a terrific class. When first asked to participate I was skeptical that an undergraduate class of mostly freshman and sophomores would be able to follow my standard research seminar (as advised by the class instructor I made very few adjustments). However, even immediately after the lecture the students were asking insightful questions. Upon my return visit, the student questions revealed a surprising depth of understanding, both about the general topic and the specific methodology. Based on this experience it is clear that this course format is an outstanding strategy to introduce undergraduates to research science.”

“Teaching in this class was such a pleasure.  It was one of those rare moments in a faculty member’s life where the pay-off far exceeds the amount of work put in.  It renewed my faith in students.  Teaching a large undergraduate course, I tend to interact with those students who are more motivated by their grades than the science.  Talking with these students and hearing their excitement was a refreshing reminder of the reward of teaching.”

“I participated in the BR 5HB Class that Dr. Ira Clark directs for two separate years and both times I was very impressed with the students and their ability to follow my research and ask great questions. It was clear from the Q&A that the student's knowledge had increased greatly from the time that I first presented my seminar to the class. It is very impressive the level of coaching and teaching that the students received from Dr. Clark. A big plus for me was being introduced to very talented students, one of which has been working in my lab for the past year and is doing great work towards a publication.”

“The level of discussion during our class meetings was very sophisticated, especially for an undergraduate course consisting of freshmen and sophomores who were new to research concepts and methods at the outset of the course.  Students had prepared specific and insightful questions related to experimental approach, design, and rationale.  It was clear that the seminar deconstruction methods and the course instructors’ guidance contributed to a depth of understanding that is not typical for undergraduates who are making their first foray into primary research literature.  Discussions related to the ‘big picture’ were most impressive since students made important connections between the main paper/lecture and other published works.  Our discussions then extended to ethical considerations as well as patent and intellectual property issues.  For the last ten years, I’ve instructed a seminar course that is part of an honors curriculum for senior undergraduate students in … majors.  Despite their extensive research laboratory experience, these upper classmen struggle with comprehension of seminar presentations and published papers, suggesting that they haven’t acquired the skills needed to extract concepts presented in a seminar or published paper.  In other words, undergraduate participation in journal clubs and research group meetings in their own laboratories appears to be inefficient in providing students with the tools for seminar/research deconstruction.   Dr. Clark’s focused approach is highly effective at providing students with these invaluable skills that they clearly are not receiving from other courses or research experiences.”
